# Supplementary material for: Macroscopic conductivity of aqueous electrolyte solutions scales with ultrafast microscopic ion motions
Source: Nat Commun. 2020 Mar 31;11:1611. doi: 10.1038/s41467-020-15450-2 (PMC7109088; doi:10.1038/s41467-020-15450-2)
Supplement: Supplementary file 1 — Supplementary Information [file 41467_2020_15450_MOESM1_ESM.pdf]

Supplementary Information

Macroscopic conductivity of aqueous electrolyte solutions scales with ultrafast microscopic ion motions

Balos et al.

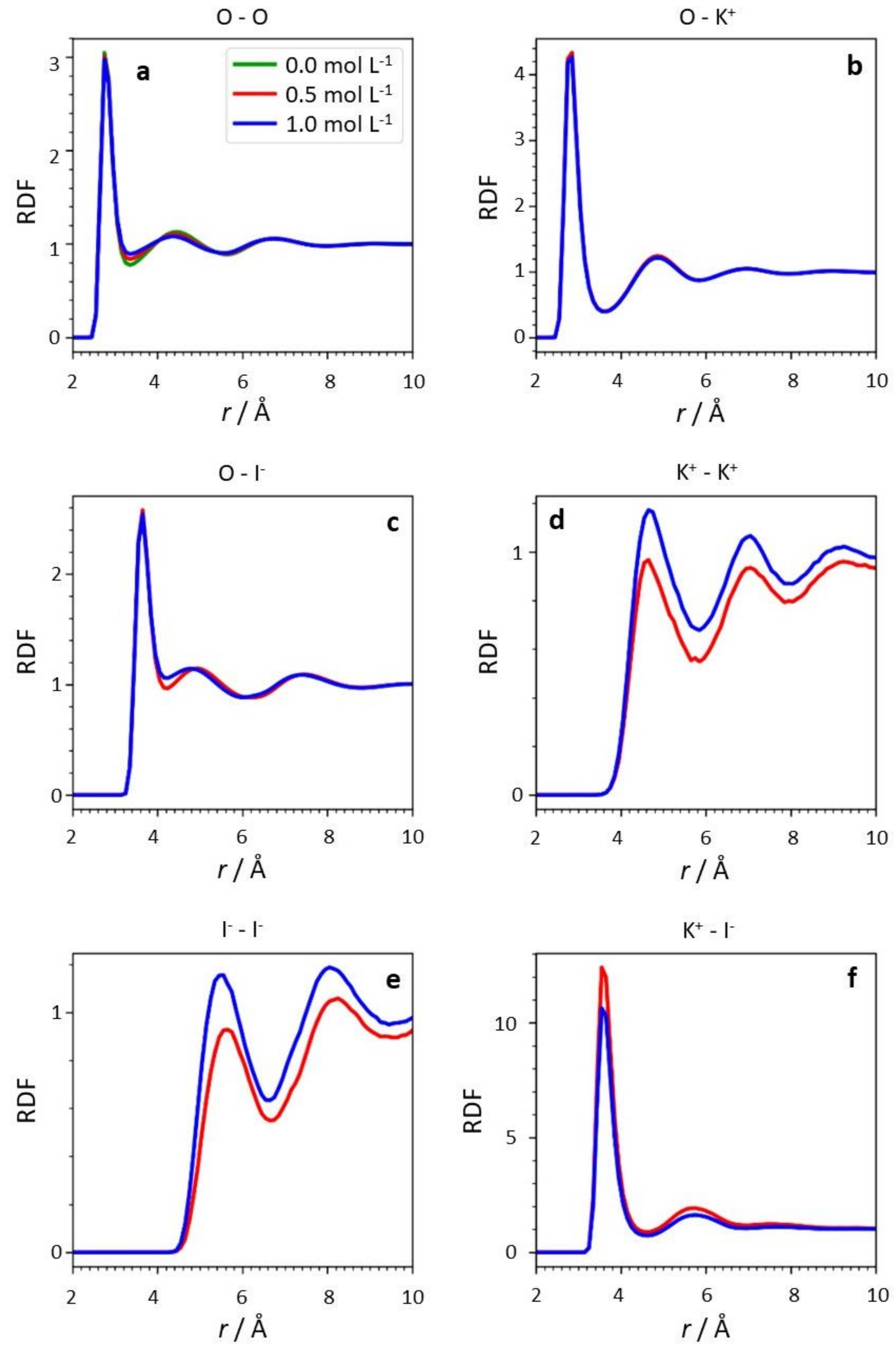

Supplementary Figure 1. Radial distribution functions (RDFs) of aqueous solutions of KI. RDFs of neat water (green), 0.5 mol L^{-1} KI (red), and 1.0 mol L^{-1} KI (blue). Panels (**a**) - (**f**) depict RDFs of water oxygen-water oxygen, water oxygen-potassium ion, water oxygen-iodide ion, potassium ion-potassium ion, iodide ion-iodide ion, and potassium ion-iodide ion, respectively.

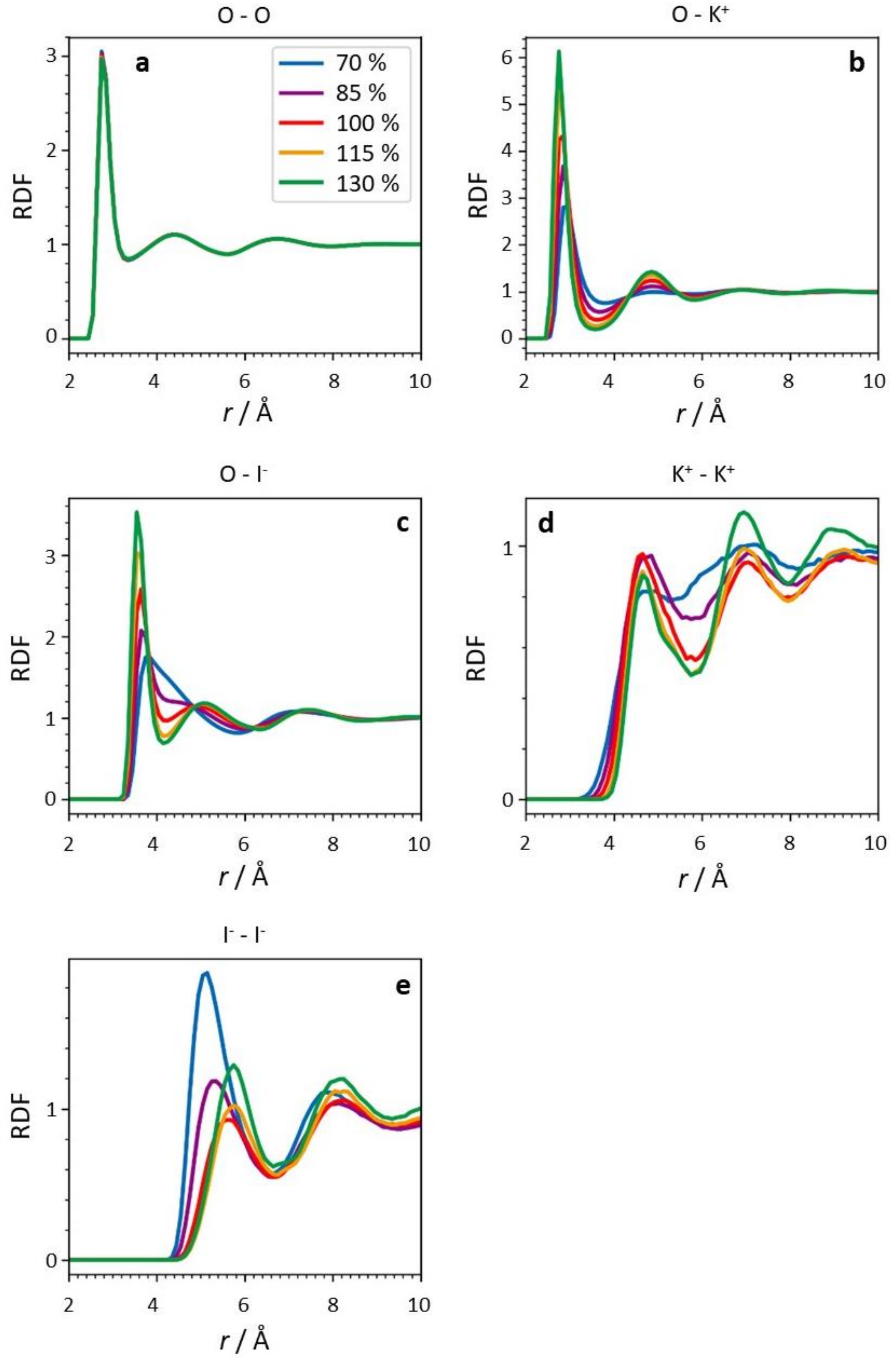

Supplementary Figure 2. Radial distribution functions (RDFs) of charge scaled systems. Panels (**a**) - (**e**) depict RDFs of 0.5 mol L^{-1} KI solutions for water oxygen-water oxygen, water oxygen-potassium ion, water oxygen-iodide ion, potassium ion-potassium ion, iodide ion-iodide ion, respectively. Blue, purple, red, orange, and green lines represent charge scaling of 70 %, 85 %, 100 % (original parameter), 115 %, and 130 %. Note that RDFs of K^+-I^- are shown in the main text (Fig. 2).

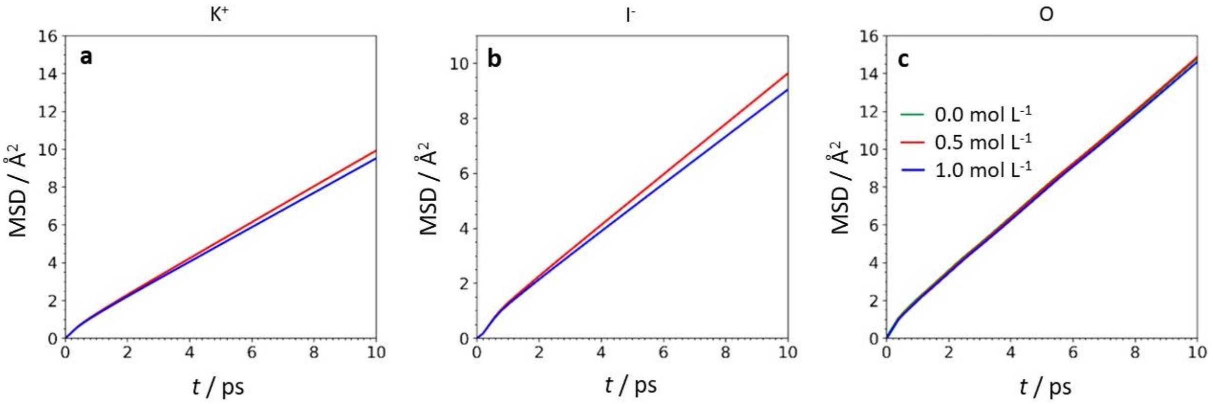

Supplementary Figure 3. Mean square displacements (MSD) of aqueous solutions of KI. MSD of (**a**) potassium ion, (**b**) iodide ion, and (**c**) water oxygen calculated from neat water (green), 0.5 mol L^{-1} KI (red), and 1.0 mol L^{-1} KI (blue).

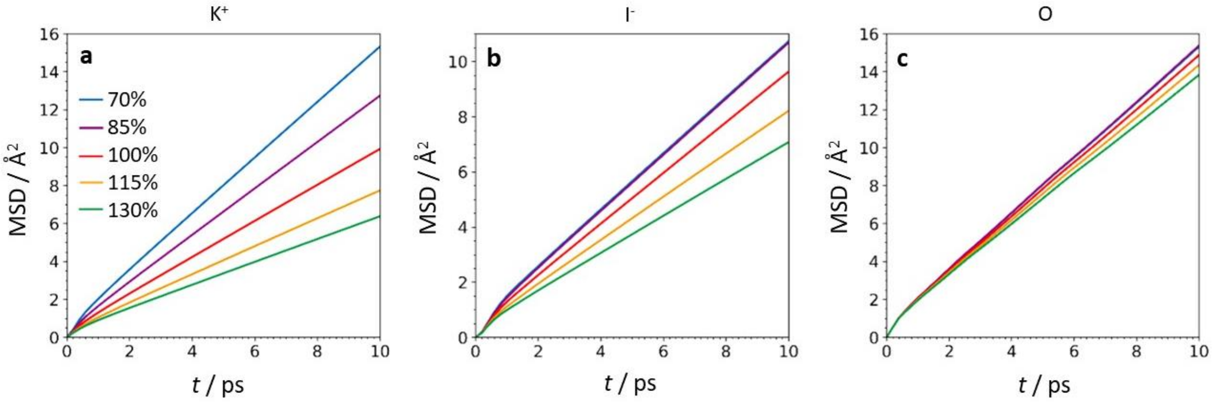

Supplementary Figure 4. Mean square displacements (MSD) of charge-scaled systems. MSD of (**a**) potassium ion, (**b**) iodide ion, and (**c**) water oxygen calculated from charge scaled simulations. Blue, purple, red, orange, and green lines represent charge scaling of 70 %, 85 %, 100 % (original parameter), 115 %, and 130 %, respectively.

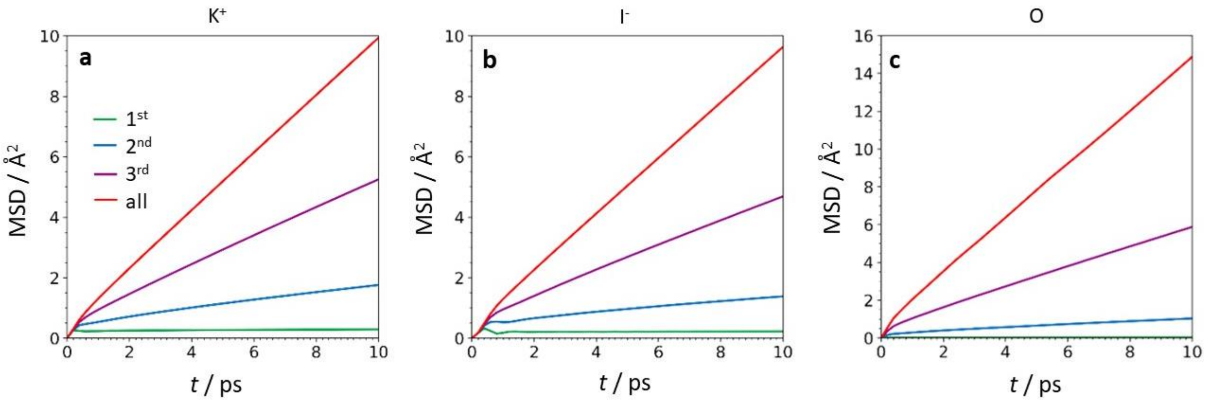

Supplementary Figure 5. Mean square displacements (MSD) of fixed-NVE simulations. MSD of (**a**) potassium ion, (**b**) iodide ion, and (**c**) water oxygen calculated from the fixed-NVE simulations. Green, blue, and purple lines represent simulations with molecules beyond the 1st, 2nd, and 3rd coordination shell constrained, respectively. The red line shows results without constraints.

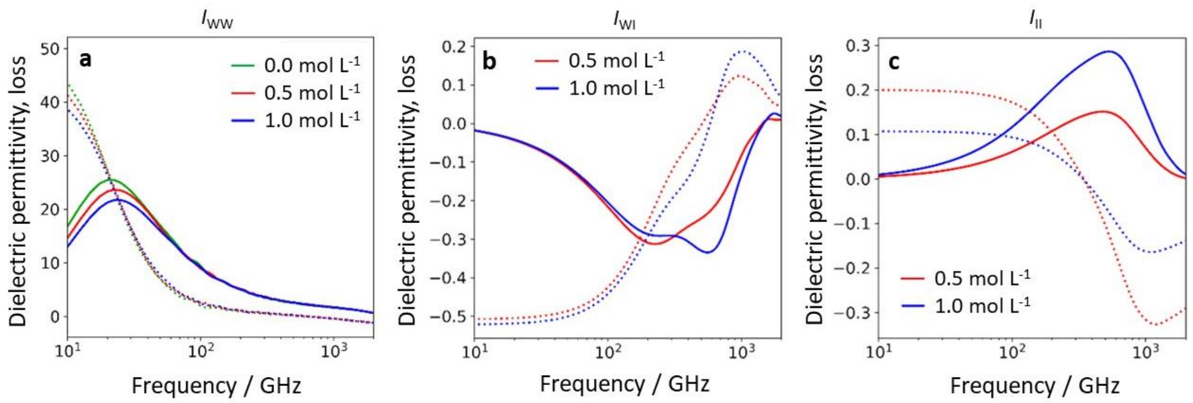

Supplementary Figure 6. Water and ion contributions to the dielectric spectra of aqueous KI solutions. I_{WW} (**a**), I_{WI} (**b**), and I_{II} (**c**) spectra of neat water (green lines), 0.5 mol L^{-1} KI (red lines), and 1.0 mol L^{-1} KI (blue line) as a function of frequency ν (=$\omega/2\pi$). Solid and dotted lines represent imaginary and real parts, respectively.

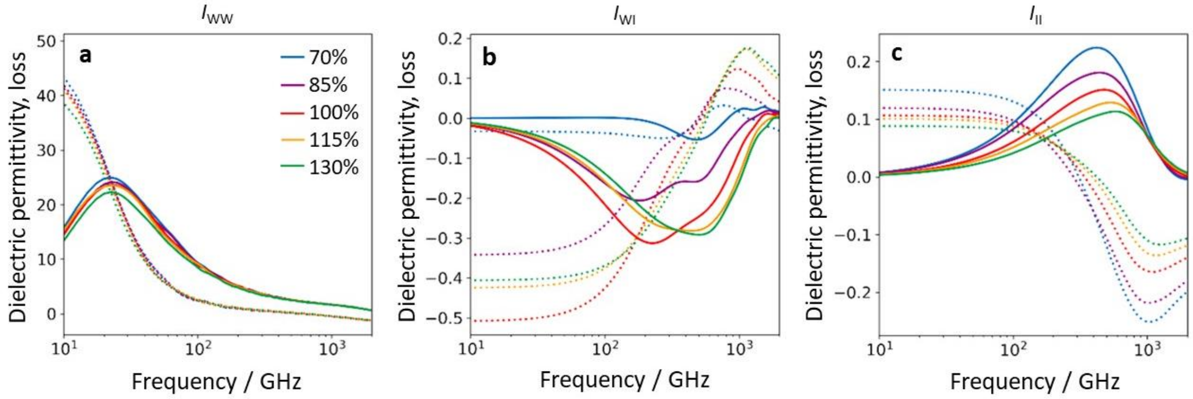

Supplementary Figure 7. Water and ion contributions to the dielectric spectra of the charge-scaled systems. I_{WW} (**a**), I_{WI} (**b**), and I_{II} (**c**) spectra of charge scaled system as a function of frequency ν (=ω/2π). Solid and dotted lines represent imaginary and real parts, respectively. Blue, purple, red, orange, and green lines represent charge scaling of 70 %, 85 %, 100 % (original parameter), 115 %, and 130 %. Note that we scale the charges to obtain different charge distributions (RDFs) in Figure 2c and Supplementray Figure 2. To isolate the effect of the distribution of the ions, we calculate the dielectric spectra (Fig. 2a and Supplementary Figure 7) assuming ions with unity charges.

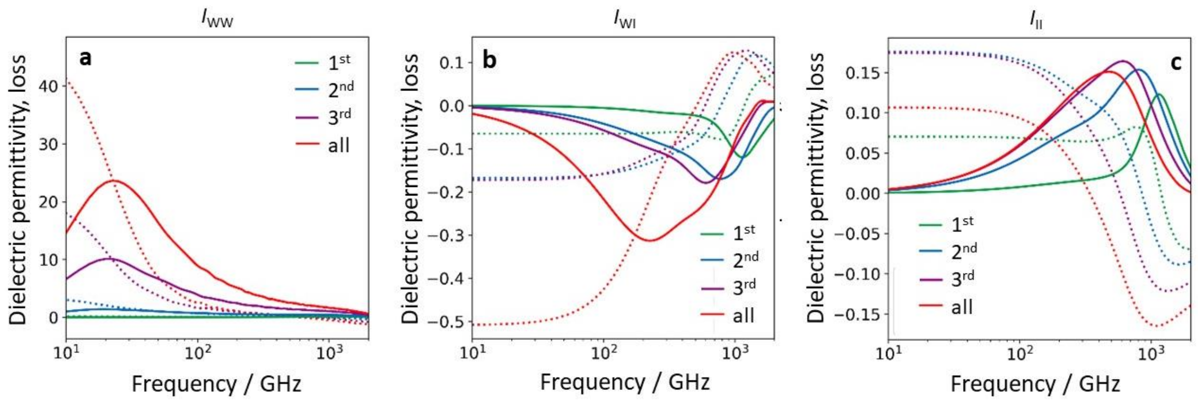

Supplementary Figure 8. Water and ion contributions to the dielectric spectra of the fixed-NVE systems. I_{WW} (**a**), I_{WI} (**b**), and I_{II} (**c**) spectra of constrained simulations as a function of frequency ν ($=\omega/2\pi$). Solid and dotted lines represent imaginary and real parts. Green, blue and purple lines represent simulations with molecules beyond the 1st, 2nd, and 3rd coordination shell constrained, respectively. The red line shows results without constraints.

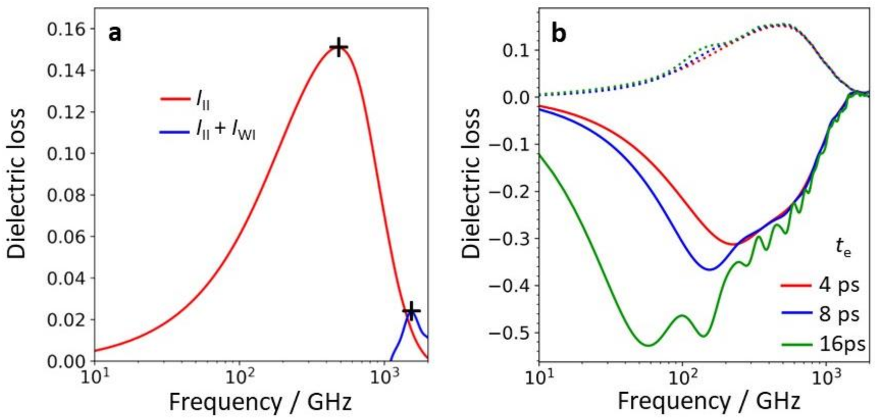

Supplementary Figure 9. Ions' contribution to the dielectric spectra. (**a**) Comparison of the ion-related polarization dynamics I_{II} and $I_{II} + I_{WI}$ for the 0.5 mol L^{-1} KI solution. Crosses indicate the location of the maxima used in Supplementary Figure 21. (**b**) I_{WI} (solid lines) and I_{II} (dotted lines) spectra for the 0.5 mol L^{-1} KI solution as a function of frequency ν (=$\omega/2\pi$) at different cut-off times, t_e.

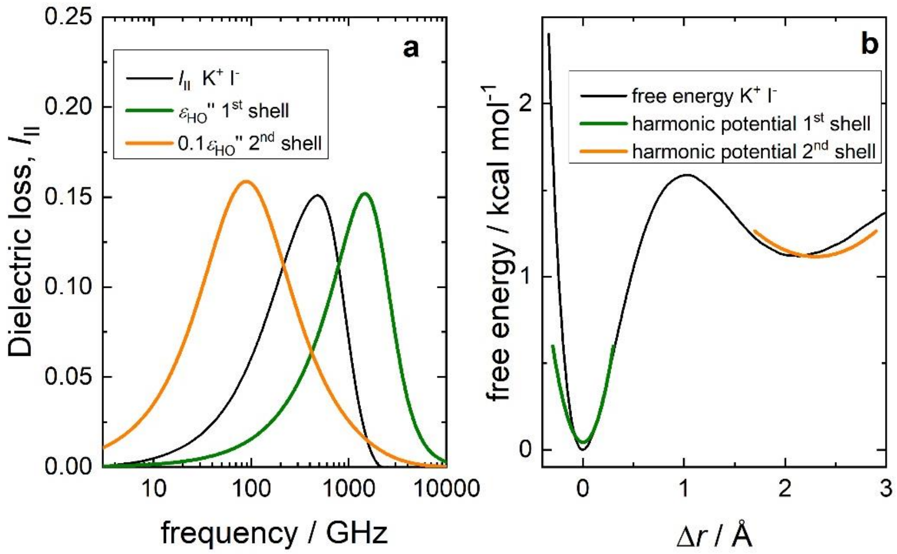

Supplementary Figure 10. Harmonic oscillator model for the second coordination shell. Dielectric loss (**a**) and potential of mean force (**b**) for 0.5 mol L^{-1} KI as obtained from molecular dynamics simulations (solid black lines) together with the harmonic oscillator model for the first coordination shell (green solid line) and the second coordination shell (orange solid line). The data shown as black and green lines are the same as in Figs. 2a and 2c of the main manuscript.

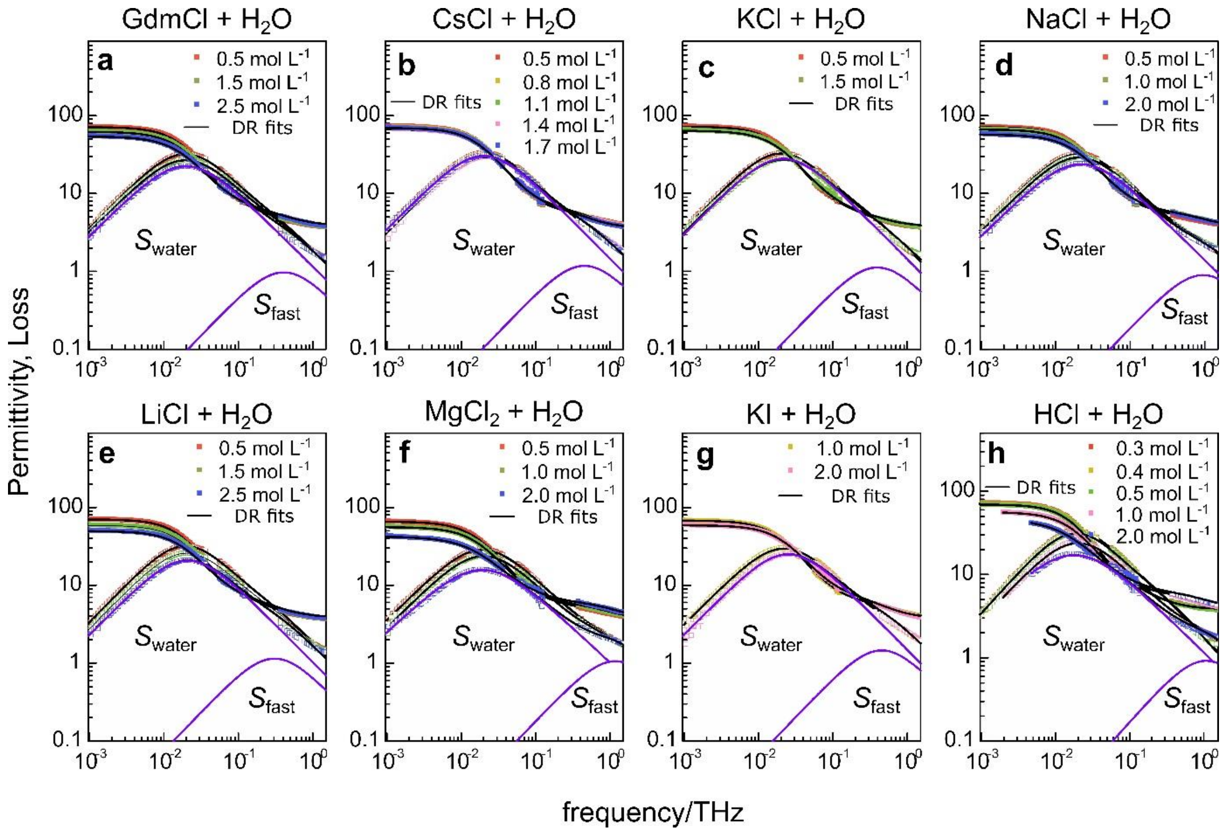

Supplementary Figure 11. Dielectric spectra of aqueous salt solutions. Spectra for solutions of (**a**) GdmCl, (**b**) CsCl, (**c**) KCl, (**d**) NaCl, (**e**) LiCl, (**f**) $MgCl_2$, (**g**) KI, and (**h**) HCl. Symbols show experimental data, solid lines correspond to fits using eq. 1 of the main manuscript. The shaded areas show the contribution of the two relaxations (shaded-blue: water, shaded red: fast) to the dielectric loss of the spectrum of the most concentrated sample. Note that for visual clarity, the conductivity contribution has been subtracted (last term of eq. 1).

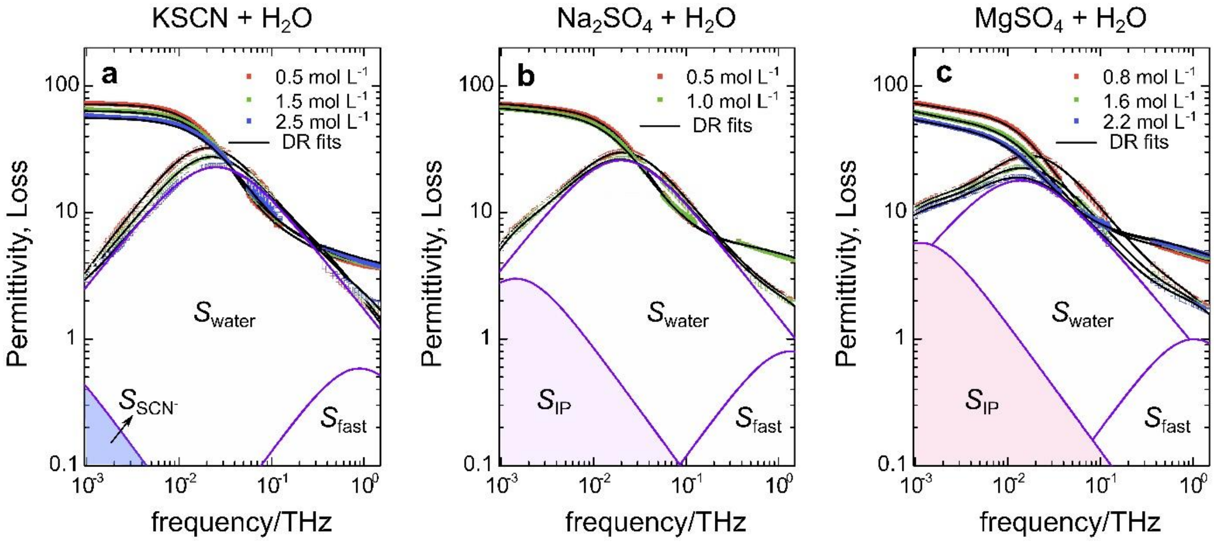

Supplementary Figure 12. Dielectric spectra of aqueous salt solutions with an additional solute relaxation. Spectra of (**a**) KSCN, (**b**) Na_2SO_4, and (**c**) $MgSO_4$. Symbols show experimental data, solid lines correspond to fits using Supplementary Equation 20. The shaded areas show the contribution of the three relaxations (shaded light blue: water, shaded red: fast, shaded dark blue: SCN^-, shaded violet: Na_2SO_4 ion-pairs, shaded brown: $MgSO_4$ ion-pairs) to the dielectric loss of the spectrum of the most concentrated sample. Note that for visual clarity, the conductivity contribution has been subtracted (last term of Supplementary Equation 20).

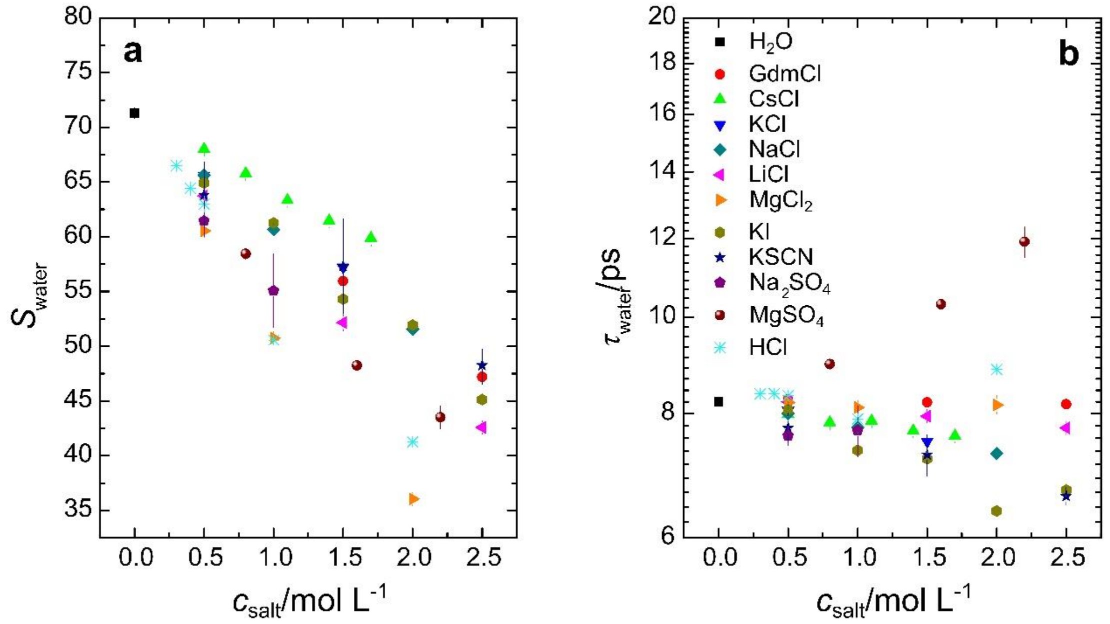

Supplementary Figure 13. Water relaxation strength and relaxation time. Values of (**a**) the bulk water relaxation amplitude, S_{water} and (**b**) the bulk water relaxation time, τ_{water}, as a function of salt concentration. The error bars correspond to the standard deviation within six independent measurements.

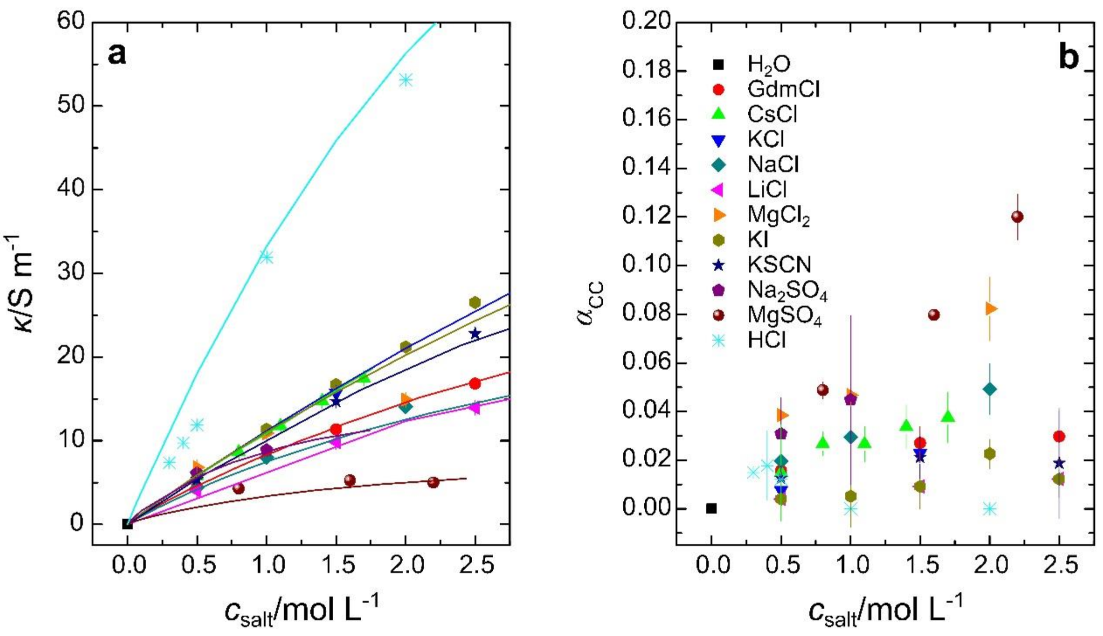

Supplementary Figure 14. Sample conductivity and Cole-Cole parameter of water relaxation. Values of (**a**) conductivity, *κ*, and (**b**) Cole-Cole parameter, α_{CC}, as a function of solute concentration. The error bars correspond to the standard deviation within six independent measurements. The solid lines in panel a correspond to fits to literature values at 25 °C for HCl,[1] KCl,[2] LiCl,[2] GdmCl,[3] KI,[4] CsCl,[5], NaCl,[6] Na_2SO_4,[7] $MgCl_2$,[8] and $MgSO_4$.[9] Literature data for KSCN[10] were measured at 20 °C. The observed deviations from linearity show that besides hydrodynamic friction also ionic friction and/or ion-pairing contribute to the macroscopic conductivity.

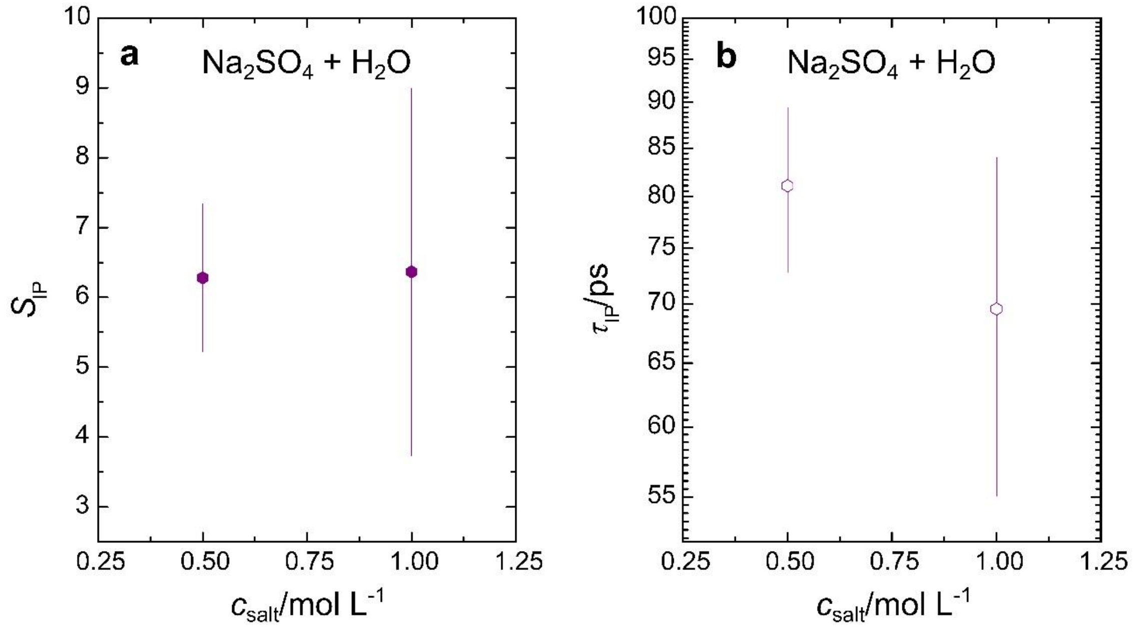

Supplementary Figure 15. Solute relaxation of Na_2SO_4. (**a**) Relaxation strength, S_{IP}, and (**b**) relaxation time, τ_{IP}, of the ion-pair relaxation as obtained by fitting Supplementary Equation 20 to the experimental spectra of solutions of Na_2SO_4. Error bars correspond to the standard deviation within six independent experiments.

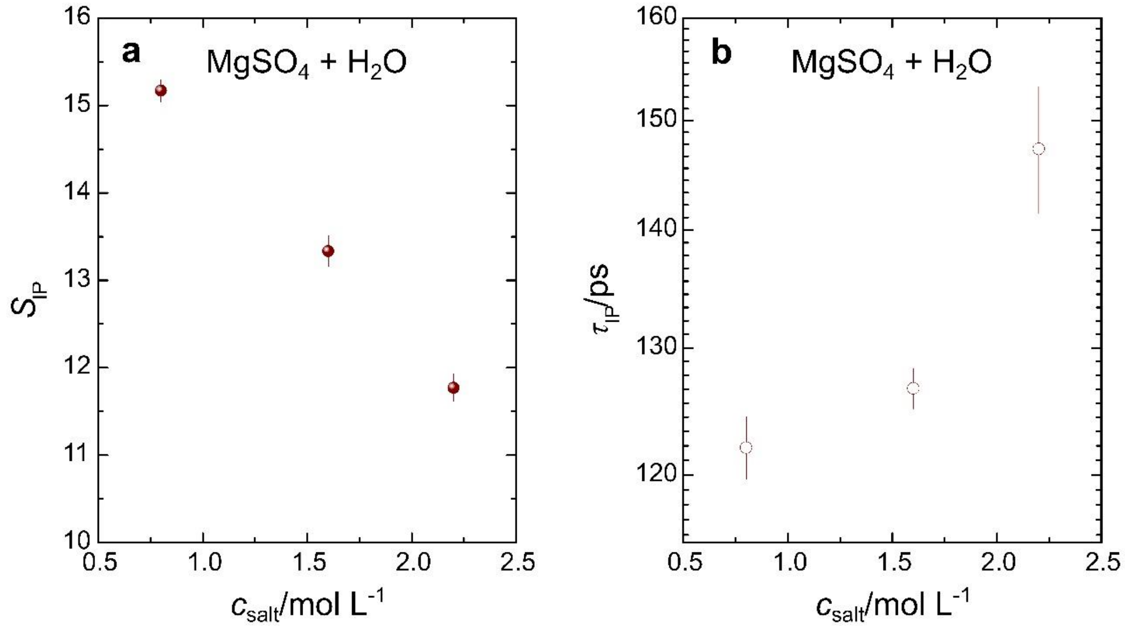

Supplementary Figure 16. Solute relaxation of $MgSO_4$. (**a**) Relaxation strength, S_{IP}, and (**b**) relaxation time, τ_{IP}, of the ion-pair relaxation as obtained by fitting Supplementary Equation 20 to the experimental spectra of solutions of $MgSO_4$. Error bars correspond to the standard deviation within six independent experiments.

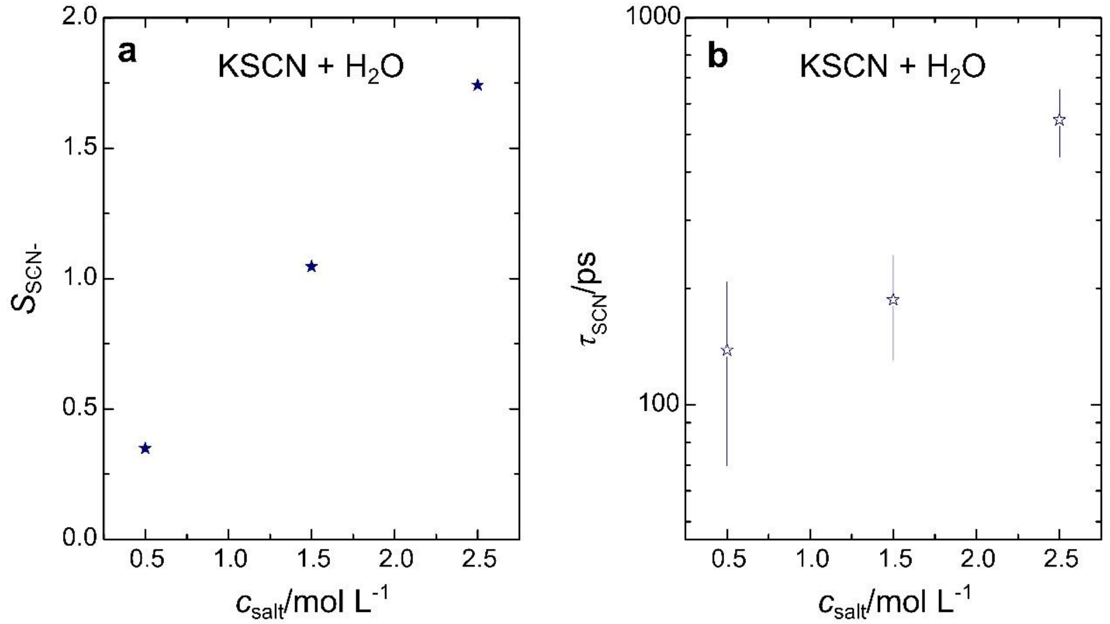

Supplementary Figure 17. Solute relaxation of KSCN. (**a**) Values of the amplitude of the SCN^- relaxation, S_{SCN^-}, calculated using Supplementary Equation 21 and (**b**) values of the relaxation time of the SCN^- relaxation as obtained from fitting Supplementary Equation 20 to the experimental data. The error bars correspond to the standard deviation within six independent measurements.

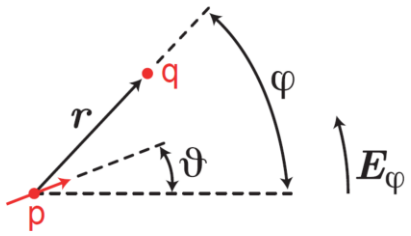

Supplementary Figure 18. Charge-dipole interaction scheme. Scheme of the dipole p interacting with an ion of charge q in an external electric field E_φ.

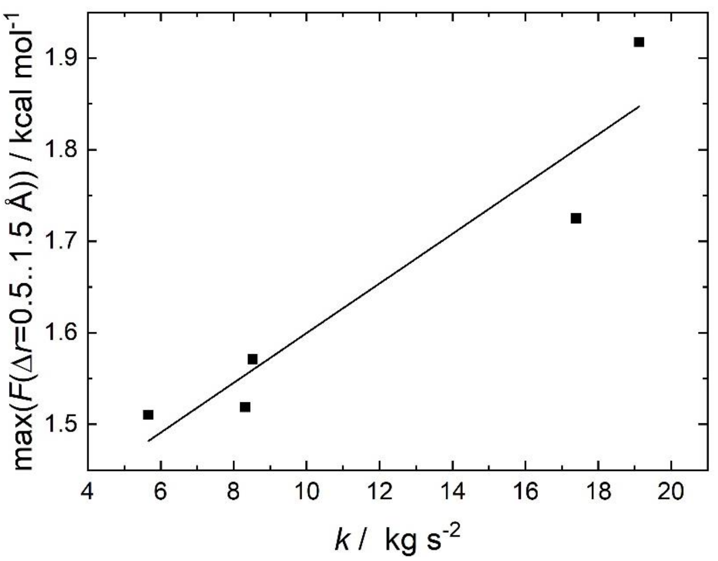

Supplementary Figure 19. Correlation between potential curvature and transport barrier. Correlation between the force constant, *k*, obtained by approximating the potential minima shown in Fig. 2c of the main manuscript by a harmonic potential with the energy barrier to escape the potential (i.e. the maximum value of $F(r)$ at $0.5 < \Delta r/\text{Å} < 1.5$, see Fig 2c of the main manuscript).

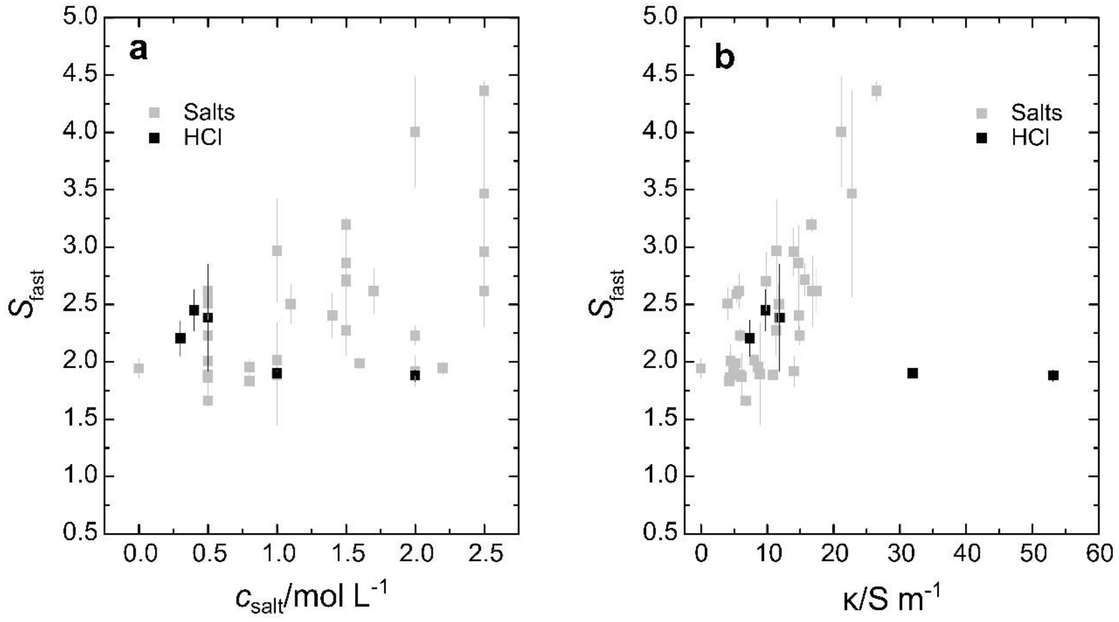

Supplementary Figure 20. Fast relaxation for solutions of HCl. Amplitudes, S_{fast}, of the fast mode as a function of (**a**) salt concentration, c_{salt}, and (**b**) conductivity, κ, for aqueous solutions of HCl (black symbols). Other salts as shown in Fig. 3 of the main manuscript are indicated as grey symbols. For high concentrations of HCl, S_{fast} does not obey the overall correlation with κ as observed for the conventional salts, which can be related to the different conduction mechanisms of the proton in aqueous solution. The error bars correspond to the standard deviation within six independent measurements.

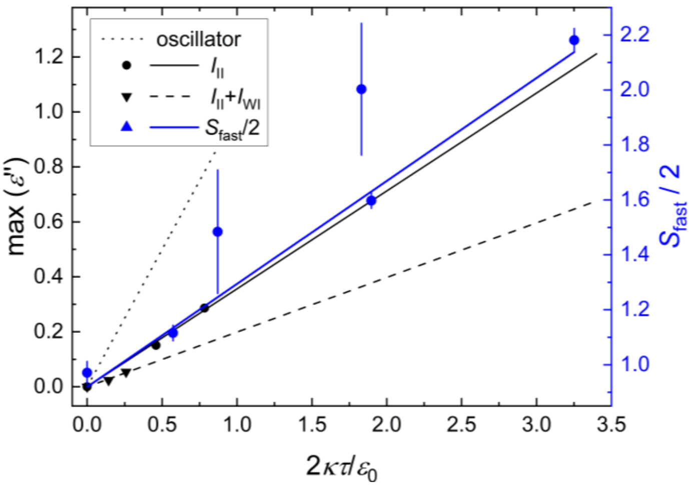

Supplementary Figure 21. Quantitative comparison of the harmonic oscillator model to MD simulations. Maximum values of $\mathrm{Im}(I_{II})$ (black circles and solid black line) and $\mathrm{Im}(I_{II} + I_{WI})$ (black triangles and dashed black line) vs. $2\kappa\tau/\varepsilon_0$ as obtained from the MD simulations for 0.5 mol L^{-1} and 1.0 mol L^{-1} KI (see Supplementary Figures 6c and 9a). Symbols show the numerical values and lines correspond to linear fits. The dotted line shows the line with unity slope as predicted by the oscillator model (Supplementary Equation 15). Blue symbols and blue solid line shows the experimental maximum of the dielectric loss of the fast relaxation, $S_{fast}/2$, for 0, 0.5, 1.0, 1.5, 2.0, and 2.5 mol L^{-1} KI. Note that the experimental values are vertically offset (see blue right-hand axis) due to the finite value of S_{fast} for neat water. The error bars correspond to the standard deviation within six independent measurements.

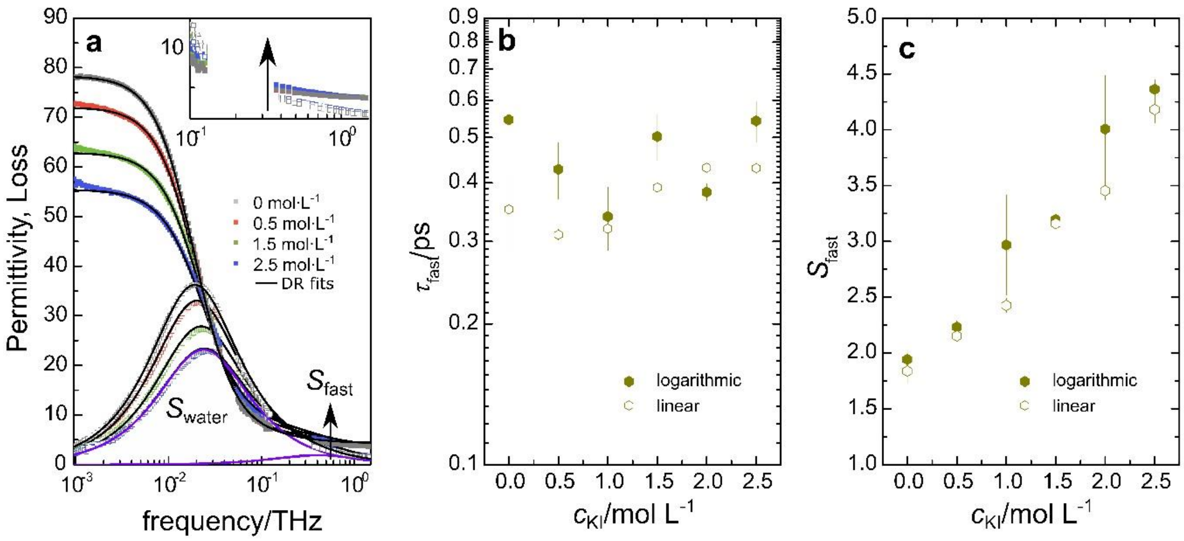

Supplementary Figure 22. Comparison between fits based on linear and logarithmic deviations. (**a**) Dielectric permittivity (filled symbols) and dielectric loss spectra (open symbols) of aqueous solutions of KI. Lines show the fits based on Supplementary Equation 22 with the relaxation model (eq. 1 of the main manuscript). The shaded areas show the contributions of the main water relaxation at 20 GHz (light blue) and the fast mode at ~ 0.3 THz (light red) to the loss spectrum of the 2.5 mol L^{-1} solution of KI. Inset shows a zoom into the 0.1 – 1.5 THz spectral range. Note that for visual clarity, the Ohmic loss (last term of eq. 1) has been subtracted. Parameters of the fast relaxation mode, τ_{fast} (**b**) and S_{fast} (**c**) obtained by using linear (filled symbols, Supplementary Equation 22) and logarithmic deviations (open symbols, Supplementary Equation 23) in the fitting procedure. The error bars correspond to the standard deviation within six independent measurements.

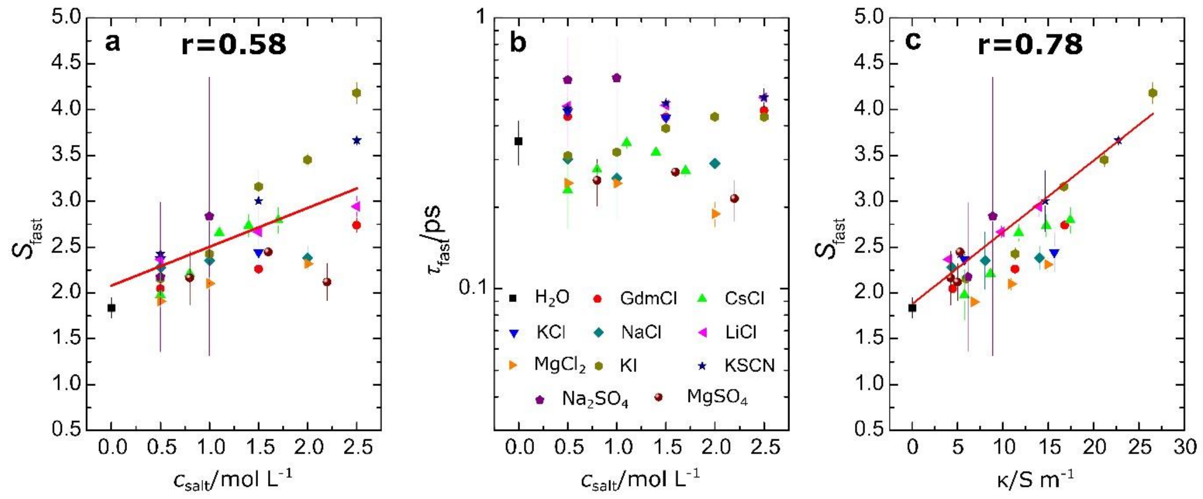

Supplementary Figure 23. Correlations based on fits using logarithmic deviations. Same as Fig. 3 of the main manuscript, except that all fits were performed based on Supplementary Equation 22, instead of Supplementary Equation S23.

Supplementary Tables

Supplementary Table 1. Simulated diffusion coefficients. Diffusion coefficient of potassium ion, iodide ion, and water oxygen in Å^2 ps^{-1}.

	Neat Water	0.5 mol/L	1.0 mol/L	charge 70%	charge 85%	charge 115%	charge 130%
K^+		0.166	0.159	0.256	0.212	0.129	0.106
I^-		0.160	0.151	0.179	0.178	0.137	0.118
O (water)	0.247	0.248	0.244	0.255	0.256	0.239	0.231

Supplementary Notes

Supplementary Note 1: Mean Square Displacements and Diffusion Coefficients

The diffusion coefficient *D* of ions as well as water molecules were obtained from the *NVE* simulations though the Einstein equation,

$$D = \frac{1}{6t}\langle |r(t) - r(0)|^2 \rangle. \quad (1)$$

The MSDs of ions and water molecules for neat water, 0.5 mol L^{-1} KI, and 1.0 mol L^{-1} KI are shown in Supplementary Figure 3 and for the charge scaled systems Supplementary Figure 4. MSDs of ions and water molecules calculated from the fixed-*NVE* simulations are shown in Supplementary Figure 5.

Supplementary Note 2: Dielectric spectra from MD simulations

The dielectric spectra were calculated through Fourier transformation of the system polarization. The total polarization of the system at time t is defined by $M(t) = \sum_i q_i r_i(t)$ where i is the atom index. Here, we use the itinerant polarization, for which r_i represents the positions of ions and water molecules that are not relocated back to the primary simulation cell.[11] The dielectric spectrum is given by

$$I(\omega) = -\frac{1}{3\epsilon_0 V k_b T}\int_{-t_e}^{t_e} dt\, e^{-i\omega t}\langle M(t)\dot{M}(0) - M(t_e)\dot{M}(0)\rangle\, f_s(t), \qquad (2)$$

where $\dot{M}(t)$ is the time derivative of $M(t)$, and V the volume of the system. t_e and $f_s(t)$ are the maximum length correlation function and a window function of Fourier transform (*vide infra*). The total polarization of the system can be decomposed into ion and water contributions, $M_{\mathrm{I}}(t) = \sum_{i\in\mathrm{ion}} q_i r_i(t)$ and $M_{\mathrm{W}}(t) = \sum_{i\in\mathrm{water}} q_i r_i(t)$. Therefore the dielectric spectra can also be decomposed into the water-water contribution $I_{\mathrm{WW}}(\omega)$, ion-ion contribution $I_{\mathrm{II}}(\omega)$, and water-ion contribution $I_{\mathrm{WI}}(\omega)$ as:[12]

$$I_{\mathrm{WW}}(\omega) = -\frac{1}{3\epsilon_0 V k_b T}\int_{-t_e}^{t_e} dt\, e^{-i\omega t}\langle M_{\mathrm{W}}(t)\dot{M}_{\mathrm{W}}(0) - M_{\mathrm{W}}(t_e)\dot{M}_{\mathrm{W}}(0)\rangle\, f_s(t), \qquad (3)$$

$$I_{\mathrm{II}}(\omega) = -\frac{1}{3\epsilon_0 V k_b T}\int_{-t_e}^{t_e} dt\, e^{-i\omega t}\langle M_{\mathrm{I}}(t)\dot{M}_{\mathrm{I}}(0) - M_{\mathrm{I}}(t_e)\dot{M}_{\mathrm{I}}(0)\rangle\, f_s(t), \qquad (4)$$

$$I_{\mathrm{WI}}(\omega) = -\frac{1}{3\epsilon_0 V k_b T}\int_{-t_e}^{t_e} dt\, e^{-i\omega t}\langle M_{\mathrm{W}}(t)\dot{M}_{\mathrm{I}}(0) - M_{\mathrm{W}}(t_e)\dot{M}_{\mathrm{I}}(0)\rangle\, f_s(t). \qquad (5)$$

For all spectral calculations, we used the Hann window function,

$$f_s(t) = \begin{cases} \cos^2\left(\frac{\pi t}{2t_e}\right) & \text{for } |t| \le t_e \\ 0 \text{ for } |t| > t_e \end{cases}. \qquad (6)$$

We set t_e to 4 ps for $I_{\mathrm{II}}(\omega)$ and $I_{\mathrm{WI}}(\omega)$ to focus on the dynamics at ~0.3 THz, whereas t_e = 60 ps was used for $I_{\mathrm{WW}}(\omega)$ and $I(\omega)$ to reproduce water relaxation at ~20 GHz. To minimize the artifact of the window function, we subtracted $M(t_e)\dot{M}(0)$ from the correlation. Imaginary and real parts of $I_{\mathrm{WW}}(\omega)$, $I_{\mathrm{WI}}(\omega)$ and $I_{\mathrm{II}}(\omega)$ are shown in Supplementary Figures 6-8.

Since the focus of the present study is the ionic dynamics at high frequencies, we use a relatively short cut-off t_e = 4 ps. As can be seen in Supplementary Figure 9, the ionic polarization components (I_{II}) are indeed rather insensitive to t_e. The ion-water contributions, I_{WI}, however, are affected by the exact value of t_e and the chosen cut-off of t_e = 4 ps does not capture contributions at lower frequencies (Supplementary Figure 9). These contributions to I_{WI} at lower frequencies typically reduce the magnitude of the dominant relaxation at ~20 GHz in the experimental spectra and represent the so-called kinetic polarization.[13] This contribution is beyond the scope of the present study and has been discussed in detail before.[14,15]

Supplementary Note 3: Harmonic oscillator model

The equation of motion for the relative distance $r(t)$ between two ions of charge $\pm q$ in the presence of a time-dependent electric field $E_r(t)$ is given by

$$\mu\ddot{r}(t) = -\gamma\dot{r}(t) - kr(t) + 2qE_r(t) \quad (7)$$

where γ is the friction coefficient and μ the reduced mass. Fourier transformation of Supplementary Equation 7 yields

$$-\mu\omega^2\tilde{r}(\omega) = i\omega\gamma\tilde{r}(\omega) - k\tilde{r}(\omega) + 2q\tilde{E}_r(\omega) \quad (8)$$

Where $\tilde{\ldots}$ denotes the Fourier-transformed quantities and $\omega = 2\pi\nu$ the angular frequency. The displacement of the oscillator, $\tilde{r}(\omega)$, is thus given as:

$$\tilde{r}(\omega) = \frac{2q\tilde{E}_r(\omega)}{k-i\omega\gamma\tilde{r}-\mu\omega^2} \quad (9)$$

The polarization $\tilde{P}_r(\omega)$ of the ion-pair is given by

$$\tilde{P}_r(\omega) = q\tilde{r}(\omega) = \hat{\varepsilon}_{\mathrm{HO}}(\omega)\varepsilon_0 V\tilde{E}_r(\omega) \quad (10)$$

where V is the relevant volume. Combining Supplementary Equations 9 and 10, we obtain:

$$\hat{\varepsilon}_{\mathrm{HO}}(\omega) = \frac{2q^2}{\varepsilon_0 V(k-i\omega\gamma-\mu\omega^2)} \quad (11)$$

To derive the contribution of the damped harmonic oscillator to the experimental spectra we account for the volume density of ion-pairs, $V = 1/c_{\mathrm{salt}}$:

$$\hat{\varepsilon}_{\mathrm{HO}}(\omega) = \frac{2c_{\mathrm{salt}}q^2}{\varepsilon_0(k-i\omega\gamma-\mu\omega^2)} \quad (12)$$

The maximum of the ion-pair oscillations Supplementary Equation 12 is located at the fundamental frequency $\omega = \omega_0 = \frac{1}{\mu\sqrt{6}}\sqrt{2\mu k - \gamma^2 + (16\mu^2k^2 - 4\mu k\gamma^2 + \gamma^4)^{1/2}} \approx \sqrt{k/\mu}$, and the contribution of the oscillator to the dielectric loss (imaginary part of Supplementary Equation 12) at this frequency is given by

$$\varepsilon_{\mathrm{HO}}''(\omega_0) \approx \frac{2c_{\mathrm{salt}}q^2}{\varepsilon_0}\frac{1}{\omega_0\gamma} \quad (13)$$

Using the Einstein relation $\gamma = k_{\mathrm{B}}T/(D_+ + D_-)$ and the relation between the diffusion coefficients, D_+ and D_- and the electrolyte's dc conductivity:

$$\kappa = \frac{c_{\mathrm{salt}}q^2}{k_{\mathrm{B}}T}(D_+ + D_-) \quad (14)$$

we substitute the friction coefficient in Supplementary Equation 13 by the conductivity:

$$\varepsilon_{\mathrm{HO}}''(\omega_0) \approx \frac{2\kappa}{\omega_0\varepsilon_0} = \frac{2\kappa\tau}{\varepsilon_0} \quad (15)$$

with $\tau = 1/\omega_0$. Hence, the harmonic oscillator model predicts the amplitude of the ions' oscillations to the dielectric loss spectra to scale linearly with the macroscopic dc conductivity and the oscillation time τ.

Supplementary Note 4: Frequency of the oscillations of the second coordination shell

In the main text, we model only fluctuations in the first ionic coordination shell with the harmonic oscillator, which leads to higher peak frequencies of the oscillator compared to the simulated I_{II} spectrum. To estimate the fluctuation frequency of the second ionic coordination shell, we apply the harmonic approximation also to the second minimum in the potential of mean force at $\Delta r \approx 2$ Å in Fig. 2c. The harmonic approximation, together with the resulting contributions to the dielectric loss, are shown as orange lines in Supplementary Figure 10. The force constant of the second coordination shell is ~15 times lower as compared to the first ionic coordination shell. This reduced force together with high over-damping results in the oscillator peaking at frequencies below the simulated I_{II}.

Supplementary Note 5: Correlation of ion oscillation and water polarization

We note that similar to the harmonic oscillator model predicting I_{II} to scale with conductivity, a model for the ion-water correlations (I_{WI}) based on ion-dipole interaction predicts I_{WI} contributions to scale with conductivity:

To address contributions due to ion-water interactions to the dielectric response (I_{WI}), we consider the motion of a single ion in an electric field affecting the polarization of its hydration shell. We model the hydration shell as a number of water molecules at a fixed distance *r* from the ion. The interaction potential between the ion with charge *q* and the dipole with dipole moment *p* is given as:

$$U = \frac{qp\cos(\varphi-\vartheta)}{4\pi\varepsilon_0 r^2} \tag{16}$$

With the angles φ and ϑ as shown in Supplementary Figure 18.

We ignore the effect of the motion of the ion with respect to the background charge on the dipole moment and we exclude the effect of the electric field on the angle ϑ, because both contributions are included in the ionic and the water contributions to the dielectric response, respectively. As water molecules in the first hydration shell are typically oriented by the charge, we assume $\varphi - \vartheta \approx \pi$ for $q > 0$ and $\varphi - \vartheta \approx 0$ for $q < 0$. Hence, the coupled equation of motion for the orientation of the water molecule is given by

$$I\ddot{\vartheta} = -\zeta\dot{\vartheta} - \frac{|q|p(\varphi-\vartheta)}{4\pi\varepsilon_0 r^2} \tag{17}$$

with $\zeta = k_{\mathrm{b}}T/D_{\mathrm{r}}$ being the rotational friction coefficient and *I* being the moment of inertia of the water molecule around one of the axes perpendicular to the dipole moment.

For each water molecule in the hydration shell, the relative motion of the ion has components in *r* and φ direction. Because the motion in *r* direction does not affect the polarization, we only consider the dynamics in φ direction. Applying an electric field in φ direction, the equation of motion for the ion is

$$m_{\pm} r^2 \ddot{\varphi} = -\gamma_{\pm} r^2 \dot{\varphi} - k_{\pm} r^2 \varphi + qrE_{\varphi} \tag{18}$$

Where $k_{\pm}$is the force constant of the potential of the ion, $m_{\pm}$ the mass of the ions, and $\gamma_{\pm} = k_{\mathrm{b}}T/D_{\pm}$ the friction coefficient of the ion. Solving the equation of motion and approximating the polarization by the polarization of N_{w} water molecules in the hydration shell of an ion, we derive the following expression for the dielectric loss at the resonance frequency $\omega_0 = \sqrt{k_{\pm}/m_{\pm}}$

$$\varepsilon''_{\mathrm{IW}}(\omega_0) = -\frac{N_W \kappa}{\varepsilon_0 \omega_0}\frac{p{\omega_0}^2}{|q|r}\left[\frac{I(A-I{\omega_0}^2)-\zeta^2}{(A-I{\omega_0}^2)^2+\zeta^2{\omega_0}^2}\right] \tag{19}$$

with $A = \frac{|q|p}{4\pi\varepsilon_0 r^2}$. Using this model, we thus also find the ion-water interaction of the dielectric response to scale linearly with conductivity, κ. According to Supplementary Equation 19, the ion-water contributions also depend on the hydration of the ion (N_W and r) and on the radius and the charge of the ion (r and q).

Supplementary Note 6: Correlation between potential curvature and transition barrier to the second shell

The scaling between the ionic fluctuation amplitude and the electrolyte conductivity implies that the curvature of the potential of the first ionic coordination shell ($F(r)$ at $\Delta r \approx 0$ Å in Supplementary Figure 10) is related to the energy barrier (maximum of $F(r)$ at $\Delta r \approx 1$ Å in Supplementary Figure 10) for the ion to translate to the second coordination shell. To demonstrate this correlation, we use the same harmonic approximation $F(\Delta r) = \frac{1}{2} k \, \Delta r^2$) as in the main manuscript in the range for potentials $F <$ 0.7 kcal/mol to determine the force constant, k, for all potentials shown in Fig. 2c of the main manuscript. As can be seen in Supplementary Figure 19, the thus obtained force constant correlates with the energetic barrier between the first and second coordination shell (i.e. the maximum value of $F(r)$ at 0.5 Å $< \Delta r <$ 1.5 Å in Fig. 2c of the main manuscript).

Supplementary Note 7: Quantitative comparison of experimental relaxation and MD simulations to the harmonic oscillator model

According to the oscillator model, the maximum of the ionic contributions to the dielectric loss scales with the conductivity, κ, and the relaxation time of the ionic fluctuations, τ (Supplementary Equation 15). To quantitatively compare the prediction of the oscillator model to the results of the MD simulations and the experiments, we show in Supplementary Figure 21 max(ε'') values as a function of $2\kappa\tau/\varepsilon_0$, for which Supplementary Equation 15 predicts unity slope. To compare the oscillator model to the results of the MD simulations, we also show the corresponding values for the maximum in the imaginary parts of the contribution of the ions $\mathrm{Im}(I_{\mathrm{II}})$ and the maximum of $\mathrm{Im}(I_{\mathrm{II}} + I_{\mathrm{WI}})$ located at ω_{max}. The corresponding simulated conductivities were obtained from the simulated diffusion coefficients (Supplementary Table 1) and $\tau = 1/\omega_{max}$. As can be seen from this comparison, the slopes obtained from the MD simulations are markedly smaller (~0.36 for I_{II}, and ~0.20 for $I_{\mathrm{II}} + I_{\mathrm{WI}}$) than unity. However, the slopes from the MD simulations agree well with the experimental values: the maximum in the dielectric loss of the fast relaxation, $S_{fast}/2$ vs $2\kappa\tau_{fast}/\varepsilon_0$ for solutions of KI has a slope of ~0.3 (blue curve + symbols in Supplementary Figure 21).

This comparison shows that there is close to quantitative agreement between the simulated I_{II} and the experimentally observed increment of S_{fast}. The ions' contributions are however smaller than what is predicted by the oscillator model (Supplementary Equation 15), which can be explained by the fact that the oscillator assumes a single potential in which the ions reside, while experiments and simulations are the result of a distribution of anharmonic potentials (see e.g. Supplementary Figure 10), which spectrally broadens I_{II} as compared to the oscillator model.

Supplementary Note 8: Data analysis for solutions of KSCN, Na_2SO_4, and $MgSO_4$

For fitting the spectra of solutions of KSCN, Na_2SO_4, and $MgSO_4$ we add an additional Debye term to the relaxation model (eq. 1 of the main manuscript) to account for low-frequency relaxations of the salts:

$$\hat{\varepsilon}(\nu) = \frac{S_j}{1+2\pi i\nu\tau_j} + \frac{S_{\text{water}}}{1+(2\pi i\nu\tau_{\text{water}})^{(1-\alpha_{CC})}} + \frac{S_{\text{fast}}}{1+2\pi i\nu\tau_{\text{fast}}} + \varepsilon_\infty + \frac{\kappa}{2\pi i\nu\varepsilon_o} \qquad (20)$$

with S_j and τ_j being the relaxation strength and the relaxation time of the lower frequency salt relaxation. For Na_2SO_4 and $MgSO_4$ such low-frequency relaxations have been assigned to the rotational relaxation of long-lived ion-pairs (*j* = IP in Supplementary Equation 20).[16–18] The non-centrosymmetric SCN^- anion, has an electrical dipole moment μ_{eff,SCN^-}= (3.3 ± 0.2) D[19] and thus rotational relaxation of the SCN^- anion may also contribute to the spectra (*j* = SCN^- in Supplementary Equation 20). Since the SCN^- relaxation has a rather low amplitude, we reduced the number of the adjustable parameters by fixing the value of the SCN^- relaxation amplitude (S_{SCN^-}) to what is expected based on its molar concentration, c_{SCN^-}, using the Cavell relation:[20]

$$S_{SCN^-} = \frac{\varepsilon_s}{\varepsilon_s+(1-\varepsilon_s)/3} \cdot \frac{N_A c_{SCN^-}}{3k_B T\varepsilon_0} \cdot \mu^2_{\text{eff},SCN^-} \qquad (21)$$

where ε_s (=S_{SCN^-}+ S_{water}+S_{fast} + ε_∞) is the static permittivity, and N_A Avogadro's constant.

The thus obtained relaxation amplitudes and relaxation times for the ion-pair relaxations for solutions of Na_2SO_4, and $MgSO_4$ and for the relaxation of SCN^- obtained by fitting Supplementary Equation 20 to the experimental spectra are shown in Supplementary Figures 15-17. The other fitting parameters are contained in Fig. 3 of the main manuscript and Supplementary Figures 13 and 14.

Supplementary Note 9: Influence of fit procedure on the parameters of the fast relaxation

The large difference in relaxation strengths of the dominant bulk water relaxation and the low amplitude fast relaxation makes it challenging to reliably separate both contributions.[21] To rule out a potential bias due to a large co-variance of the parameters of both relaxations, we test two different optimization approaches. When fitting eq. 1 of the main manuscript (or Supplementary Equation 20) to the experimental spectra we optimize (i) the sum of the squared linear deviations (χ_{lin}) of the fitted values $\varepsilon'_{\text{fit}}$, $\varepsilon''_{\text{fit}}$ from the experimental values, ε'_{ex}, $\varepsilon''_{\text{ex}}$:

$$\chi_{\text{lin}} = \sum(\varepsilon'_{\text{fit}} - \varepsilon'_{\text{ex}})^2 + \sum(\varepsilon''_{\text{fit}} - \varepsilon''_{\text{ex}})^2 \tag{22}$$

or (ii) the sum of the squared logarithmic deviations (χ_{log}):

$$\chi_{\text{log}} = \sum(\log\varepsilon'_{\text{fit}} - \log\varepsilon'_{\text{ex}})^2 + \sum(\log\varepsilon''_{\text{fit}} - \log\varepsilon''_{\text{ex}})^2 \tag{23}$$

The parameters for the fast relaxation obtained from both approaches for solutions of KI are shown in Supplementary Figure 22, together with the fits on a linear scale. As can be seen from these parameters, both approaches yield similar trends for the fast relaxation. Since fits optimizing χ_{log} have intrinsically a higher weight on the high-frequency (low amplitude) spectral range at > 0.1 THz than fits based on χ_{lin}, we use fits based on logarithmic deviations (Supplementary Equation 23) throughout the main manuscript. Most importantly, as can be seen in Supplementary Figure 23, the main observation – the correlation of S_{fast} with κ, as shown in Fig. 3c of the main manuscript – is also obtained using fits based on Supplementary Equation 22.

Supplementary References

1. Haynes, W. M. *CRC Hanbook of Chemistry and Physics*. (CRC Press, 2014).
2. Della Monica, M., Ceglie, A. & Agostiano, A. Extension of the Falkenhagen equation to the conductivity of concentrated electrolyte solutions. *J. Phys. Chem.* **88**, 2124–2127 (1984).
3. Hunger, J., Niedermayer, S., Buchner, R. & Hefter, G. Are Nanoscale Ion Aggregates Present in Aqueous Solutions of Guanidinium Salts? *J. Phys. Chem. B* **114**, 13617–13627 (2010).
4. Goldsack, D. E., Franchetto, R. & Franchetto, A. Solvation effects on the conductivity of concentrated electrolyte solutions. *Can. J. Chem.* **54**, 2953–2966 (1976).
5. Chen, T., Hefter, G. & Buchner, R. Dielectric Spectroscopy of Aqueous Solutions of KCl and CsCl. *J. Phys. Chem. A* **107**, 4025–4031 (2003).
6. Buchner, R., Hefter, G. T. & May, P. M. Dielectric Relaxation of Aqueous NaCl Solutions. *J. Phys. Chem. A* **103**, 1–9 (1999).
7. Isono, T. Density, viscosity, and electrolytic conductivity of concentrated aqueous electrolyte solutions at several temperatures. Alkaline-earth chlorides, lanthanum chloride, sodium chloride, sodium nitrate, sodium bromide, potassium nitrate, potassium bromide, and cadmium nitrate. *J. Chem. Eng. Data* **29**, 45–52 (1984).
8. Friesen, S., Hefter, G. & Buchner, R. Cation Hydration and Ion Pairing in Aqueous Solutions of $MgCl_2$ and $CaCl_2$. *J. Phys. Chem. B* **123**, 891–900 (2019).
9. Calvert, R., Cornelius, J. A., Griffiths, V. S. & Stock, D. I. The Determination of the Electrical Conductivities of Some Concentrated Electrolyte Solutions Using a Transformer Bridge. *J. Phys. Chem.* **62**, 47–53 (1958).
10. Gupta, R. L. & Ismail, K. Electrical Conductance of a Mixture of Sodium and Potassium Thiocyanates in Aqueous Medium at 25 °C. *Bull. Chem. Soc. Jpn.* **61**, 2605–2608 (1988).
11. Zhang, C. & Sprik, M. Finite field methods for the supercell modeling of charged insulator/electrolyte interfaces. *Phys. Rev. B* **94**, 245309 (2016).
12. Rinne, K. F., Gekle, S. & Netz, R. R. Dissecting ion-specific dielectric spectra of sodium-halide solutions into solvation water and ionic contributions. *J. Chem. Phys.* **141**, 214502 (2014).
13. Hubbard, J. & Onsager, L. Dielectric dispersion and dielectric friction in electrolyte solutions. I. *J. Chem. Phys.* **67**, 4850–4857 (1977).
14. Rinne, K. F., Gekle, S. & Netz, R. R. Ion-Specific Solvation Water Dynamics: Single Water versus Collective Water Effects. *J. Phys. Chem. A* **118**, 11667–11677 (2014).
15. Sega, M., Kantorovich, S. & Arnold, A. Kinetic dielectric decrement revisited: phenomenology of finite ion concentrations. *Phys. Chem. Chem. Phys.* **17**, 130–133 (2015).
16. Buchner, R., Chen, T. & Hefter, G. Complexity in "Simple" Electrolyte Solutions: Ion Pairing in $MgSO_4$(aq). *J. Phys. Chem. B* **108**, 2365–2375 (2004).
17. Buchner, R., Capewell, S. G., Hefter, G. & May, P. M. Ion-Pair and Solvent Relaxation Processes in Aqueous Na_2SO_4 Solutions. *J. Phys. Chem. B* **103**, 1185–1192 (1999).
18. Mamatkulov, S. I., Rinne, K. F., Buchner, R., Netz, R. R. & Bonthuis, D. J. Water-separated ion pairs cause the slow dielectric mode of magnesium sulfate solutions. *J. Chem. Phys.* **148**, 222812 (2018).
19. Balos, V., Kim, H., Bonn, M. & Hunger, J. Dissecting Hofmeister Effects: Direct Anion-Amide Interactions Are Weaker than Cation-Amide Binding. *Angew. Chemie Int. Ed.* **55**, 8125–8128 (2016).
20. Cavell, E. A. S., Knight, P. C., Sheikh, M. A., A., S. M. & Sheikh, M. A. Dielectric relaxation in non aqueous solutions. Part 2. Solutions of tri(n-butyl)ammonium picrate and iodide in polar solvents. *Trans. Faraday Soc.* **67**, 2225–2233 (1971).
21. Vinh, N. Q. *et al.* High-precision gigahertz-to-terahertz spectroscopy of aqueous salt solutions as a probe of the femtosecond-to-picosecond dynamics of liquid water. *J. Chem. Phys.* **142**, 164502 (2015).
